# Cross-sectional study of CD4:CD8 ratio recovery in young adults with perinatally acquired HIV-1 infection

**DOI:** 10.1097/MD.0000000000009798

**Published:** 2018-02-23

**Authors:** Katrina M. Pollock, Hannah Pintilie, Caroline Foster, Sarah Fidler

**Affiliations:** a Section of Virology, Department of Medicine, Imperial College London; b Jefferiss Wing, Centre for Sexual Health, Imperial College Healthcare NHS Trust; c 900 Clinic, Imperial College Healthcare NHS Trust; d Division of Infectious Diseases, Department of Medicine, Imperial College London, London, UK.

**Keywords:** adolescent, CD4:CD8 ratio, cytomegalovirus, HIV-1, infectious disease transmission, vertical

## Abstract

Antiretroviral therapy (ART) has improved survival into adulthood for young people with perinatally acquired HIV-1 (yp-PaHIV), but long-term prognosis remains unclear. We hypothesized that on-going immune activation, reflected in the failure of CD4:CD8 ratio normalization would be observed in yp-PaHIV, despite ART.

A cross-sectional study of routinely collected clinical data from a cohort of yp-PaHIV (≥16 years).

Data were collected from records of individuals attending a specialist clinic for yp-PaHIV transitioning to adult care. CD4:CD8 ratio and proportion with CD4:CD8 ratio ≥1, demographic data and viral parameters, including HIV-1 viral load (VL) and human cytomegalovirus (CMV) IgG, were analyzed with IBM SPSS Statistics v22.

A total of 115 yp-PaHIV, median (IQR) age 22.0 (20.0–24.0) years, were studied, of whom 59 were females, and the majority were Black African 75/115 (65.2%). Where measured, CMV antibodies were frequently detected (71/74, 95.9%) and CMV IgG titre was inversely associated with CD4:CD8 ratio, (Rho −0.383, *P = .*012). Of those taking ART, 69 out of 90 (76.7%) yp-PaHIV had suppressed HIV viremia (<50 RNA copies/mL) and recovery of CD4:CD8 ratio to ≥1 was seen in 26 out of 69 (37.7%) with suppressed HIV viremia. Persistence of low CD4:CD8 ratio was observed even in those with a CD4 count ≥500 cells/μL, where 28/52 (53.8%) had a CD4:CD8 ratio <1. Of those with suppressed viremia, the median (IQR) age for starting ART was 8.0 (5.0–12.8) years and CD4:CD8 ratio was inversely associated with age at ART start, Rho −0.348, (*P = .*028).

In this cohort of yp-PaHIV, despite lifelong HIV infection and widespread CMV coinfection, CD4:CD8 ratio recovery rate was comparable to adults treated in acute infection. Where persistence of CD4:CD8 ratio abnormality was observed, on-going immune activation may have significance for non-AIDS outcomes. Taken together our findings indicate immune resilience to be a feature of these adult survivors of perinatally acquired HIV infection, which can be supported with early antiretroviral therapy.

## 1 Introduction

Young people living with perinatally acquired HIV infection (yp-PaHIV) are unique in having experienced lifelong HIV infection, but little is known of the long-term prognosis. World Health Organization (WHO) guidelines recommending combined antiretroviral therapy (ART) regardless of age, CD4 cell count or WHO disease stage will likely increase longevity, reflecting global trends.^[1–5]^ Longer treatment duration, exposure to antiretroviral medication and ageing of these individuals requires new approaches for assessing treatment response and prognosis.

It has been hypothesized that HIV infection is associated with premature or accelerated biological ageing, even when viremia is suppressed. Reports of non-AIDS morbidities associated with aging occurring more frequently and prematurely in persons living with HIV infection (PLWH) compared with the general population mean this question deserves attention.^[6–8]^ Chronic immune activation may constitute an important component of the mechanism underlying these observations. Research in this area should meet the need for clinically validated biomarkers to identify those at risk, despite suppressive ART.

The CD4:CD8 ratio could serve as such a clinical biomarker in treated individuals.^[9]^ Data on CD4:CD8 ratio recovery to normal levels (≥1) in children and young adults with perinatally acquired HIV infection are limited. The CD4:CD8 ratio was ≥1 in more than half of children and adolescents in one study. This could indicate better recovery than has been reported in adults and this recovery may continue with years on ART.^[10,11]^ The cause of chronic immune activation in treated HIV infection is unclear but the relationship with the HIV reservoir may have implications for cure strategies (reviewed in ^[12]^). Young adults living with perinatally acquired HIV infection are unusual in having lifelong infection with a CD4 tropic virus acquired prior to immune maturation. The study of these individuals presents a unique opportunity to identify those with more or less favourable immune adaptation to chronic HIV infection. Such an approach could provide insights into the impact, monitoring, and treatment of immune activation and conversely into favorable adaptation important for HIV cure research.

Our objective was to measure the frequency of those with or without CD4:CD8 ratio recovery in a cohort of yp-PaHIV. We hypothesized that a potential biomarker of immune activation (CD4:CD8 ratio <1) would be observed in yp-PaHIV despite viral suppression. Here we propose that CD4:CD8 ratio might be a more sensitive tool than CD4 count alone to identify those at risk and those who conversely, through CD4:CD8 ratio recovery, might have better immune resilience.

## 2 Methods

### 2.1.1 Study design and setting

A cross-sectional study of CD4:CD8 ratio recovery in young adults with perinatally acquired HIV infection attending a specialist clinic was performed.

### 2.1.2 Data collection

Data were accessed from the electronic records from the Imperial College Healthcare NHS Trust, London, UK “900 clinic” on July 9, 2015. All records of individuals attending the clinic at this time were included. This clinic provides outpatient HIV care for adolescents and young adults aged 16 years and over with perinatally acquired HIV infection. Demographic data including age, gender, ethnicity, and ART regimen were collected. CD4:CD8 ratios were calculated using absolute CD4 and CD8 cell count values reported by the local accredited Clinical Pathology laboratory. Human cytomegalovirus (CMV) data (qualitative and quantitative IgG levels) were retrieved from separate electronic records and matched to the main database. CMV testing had been conducted according to clinical indication. Participants with suppressed HIV viremia were defined as those with HIV viral load (VL) <50 RNA copies/mL on most recent testing for routine clinical monitoring.

### 2.1.3 Data analysis

The anonymized data were coded and analyzed using (IBM) SPSS version 22. Statistical analysis followed tests for normality of the data distribution. For the purposes of analysis, low CD4 count was defined as <500 cells/μL and normal CD4 as ≥900 cells/μL. CD4:CD8 ratio recovery was defined as a ratio of ≥1. Two independent variables were compared using Mann-Whitney *U* testing and correlation between 2 continuous variables using Spearman's rank coefficient. Fishers exact 2-sided test was used to analyze 2 by 2 contingency tables as indicated. A value of *P*<.05 was deemed significant. Where data were unavailable for the whole cohort, for example, CMV coinfection data, separate subgroup analyses were conducted.

### 2.1.4 Ethical review

Data were made available to members of the clinical care team and anonymized prior to analysis in accordance with National Research Ethics Service guidance on using routinely acquired clinical data for research purposes (www.hra.nhs/uk).

## 3 Results

### 3.1 Characteristics of cohort studied

Individuals with perinatally acquired HIV infection (n = 115) who had records available at the clinic at the time of sampling were included. The cohort was equally distributed between females (n = 59) and males (n = 56) and the majority were Black African, (75/115, 65.2%). The median (IQR) age of the cohort was 22.0 (20.0–24.0) years. Hepatitis B coinfection (positive hepatitis B surface antigen result) and hepatitis C coinfection (positive hepatitis C IgG result) were infrequently observed (Table 1). Ninety out of 115 (78.3%) yp-PaHIV were prescribed ART, of whom 69 out of 90 (76.7%) had suppressed HIV viremia.

**Table 1:**
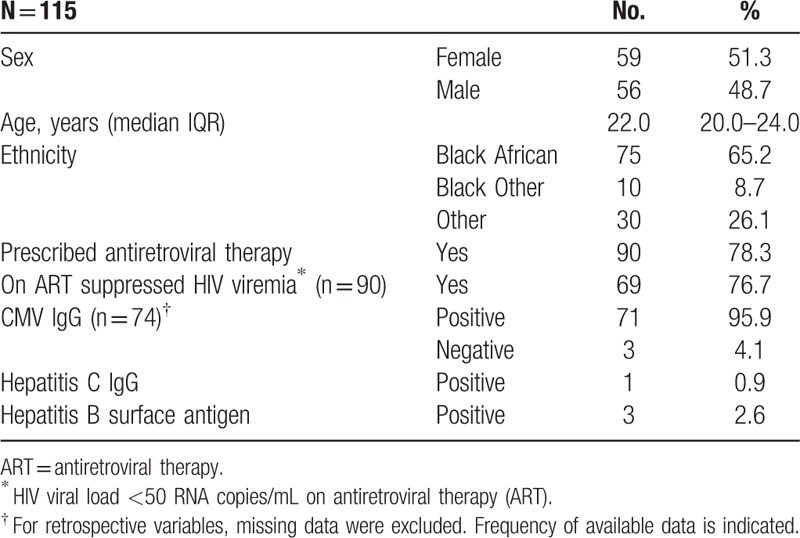
Demographics of cohort.

### 3.2 Impact of antiretroviral therapy and suppression of HIV viremia on CD4:CD8 ratio recovery

In those prescribed ART (n = 90), suppression of HIV viremia to <50 RNA copies/mL was associated with CD4:CD8 ratio recovery to ≥1 (*P = .*003). The number of years since first recorded suppression of HIV VL was median (IQR) 11.0 (8.0–15.3) years in individuals where data were available (n = 26). Of 69 individuals on ART with suppressed HIV VL, the majority, n = 43 (62.3%) had a persistently low CD4:CD8 ratio <1. Twenty-six individuals on ART with suppressed HIV VL had CD4:CD8 ratio recovery to ≥1; the overall recovery rate of CD4:CD8 ratio to ≥1 was therefore 37.7%. There was no association of the prescribed ART regimen containing a boosted protease inhibitor (n = 40) versus no boosted protease inhibitor, with CD4:CD8 ratio recovery to ≥1, (*P = .*802).

### 3.3 Impact of CMV coinfection on CD4:CD8 ratio recovery

**Figure 1:**
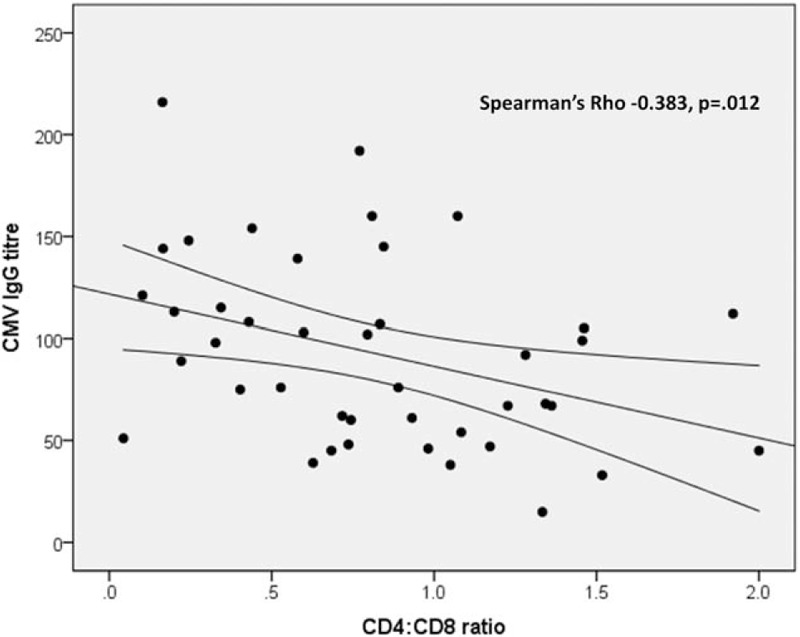
Graph shows inverse correlation of CMV IgG titre (where measured, n = 42 individuals) with CD4:CD8 ratio, Spearman's Rho = −0.383 (*P = .*012). Linear fit line with 95% confidence interval is displayed. CMV IgG was measured according to clinical indication on a case-by-case basis. CMV = cytomegalovirus.

A high prevalence of CMV coinfection was observed; where data were available (n = 74), 71 out of 74 individuals (95.9%) had a positive CMV IgG result documented. Where CMV IgG titre was recorded (measured in the Clinical Pathology Laboratory; n = 42 individuals) there was a negative correlation with CD4:CD8 ratio (Rho = −0.383, *P = .*012) (Fig. 1). There was also a negative correlation of CMV IgG titre with the lowest CD4 cell count measured in early adulthood (age 16+ years) following transfer to the yp-PaHIV clinic (Rho = −0.518, p<.001). Strong correlation between the dates of these 2 measurements indicated they were contemporaneous (Rho = 0.428, *P = .*003).

### 3.4 Association of CD4+ cell compartment recovery with CD4:CD8 ratio recovery

Among those on ART with suppressed HIV VL and where records were available (n = 35), median (IQR) nadir CD4 count was 280 (138–435) cells/μL and 52 of those on ART with suppressed viremia (n = 69) had CD4 count recovery to ≥500 cells/μL, (75.4%). Of these just over half had a persistently low CD4:CD8 ratio <1 (28/52, 53.8%) and the remainder, n = 24 had a CD4:CD8 ratio ≥1 (24/52, 46.2%). Persistence of low CD4:CD8 ratio was observed in those few achieving a CD4 count ≥900 cells/μL (n = 15), where 7 out of 15 (46.7%) had a CD4:CD8 ratio <1. Most of those individuals with a CD4 count <500 cells/μL (n = 17) had a persistently low CD4:CD8 ratio <1 (15/17, 88.2%). Those with a CD4 count <500 cells/μL were therefore more likely to have a persistently low CD4:CD8 ratio <1 than those with a CD4 count ≥500 cells/μL (*P = .*019).

### 3.5 Association of age, age at starting ART and gender with CD4:CD8 ratio recovery

There was a negative association of CD4:CD8 ratio with age, (Rho = −0.245 *P = .*042) amongst individuals on ART with suppressed HIV VL (n = 69). Of these and where records were available (n = 35), median (IQR) time on ART (n = 40) was 13.0 (9.0–17.0) years. Of those with suppressed HIV viremia, median (IQR) age at ART start was 8.0 (5.0–12.8) years, where data were available (n = 40). In this group, CD4:CD8 ratio correlated inversely with age at which ART was started (Rho −0.348, *P = .*028). In those on ART with suppressed viremia (n = 69), the median (IQR) age of females was 23.5 (20.0–25.0) years and males was 22.0 (20.0–24.0) years. We found no differences by gender in CD4:CD8 ratio recovery and the median CD4:CD8 ratio was <1 for both males and females.

## 4 Discussion

We present data from a small but unique cohort of young adults with perinatally acquired HIV infection surviving into adulthood. In accordance with the study hypothesis, persistent immune activation, reflected in the failure of CD4:CD8 ratio normalization, was observed in the majority (62.3%) of yp-PaHIV despite ART. The rate of CD4:CD8 ratio recovery was therefore 37.7% in those with suppressed HIV viremia. This was better than expected given the challenge to immune recovery experienced in this cohort of yp-PaHIV; including lifelong HIV infection, widespread CMV coinfection (>95%) and a median delay of eight years after birth prior to ART initiation. This rate of CD4:CD8 ratio recovery was comparable to levels reached amongst adults treated in acute HIV infection and better than for adults starting ART in chronic infection, where a range of recovery rates have been reported that are frequently less than 20%.^[13–16]^

The high rate of persistent CD4:CD8 ratio abnormality despite ART that we report may have significance for future non-AIDS morbidity and mortality, including diseases associated with ageing in yp-PaHIV. This abnormality was observed despite CD4 count recovery to normal or near normal levels (≥900 or ≥500 cells/μL), indicating CD8+ T-cell compartment abnormality. Failure of CD4+ T-cell homeostasis harbingers widespread immune dysregulation in untreated HIV infection ^[17–19]^ and even with ART-induced recovery, CD8+ T-cell activation can persist (reviewed in ^[20]^). Incomplete restoration of the CD4:CD8 ratio with ART in children and adults is associated with immunological abnormalities including lower frequencies of naive CD4+ and CD8+ T-cells and activated or senescent T-cell phenotypes and poor prognosis with an increased risk of non-AIDS morbidity and mortality.^[11,21–23]^

The mechanism of on-going immune activation in treated HIV infection has not been elucidated, although the coinfecting pathogen CMV, which was highly prevalent in this cohort of individuals from a predominantly Black African background, is likely to play a role.^[24–27]^ The high rate of positive CMV serology we observed was likely due to infection acquired in childhood.^[28]^ CMV coinfection in adults has been associated with activation of CD8+ T-cells and reduced CD4:CD8 ratio normalization compared with uninfected individuals .^[27,29,30]^ The inverse relationship of CD4:CD8 ratio with CMV IgG titre in this cohort indicated an association with chronic immune activation.^[28]^

CD4:CD8 ratio correlated inversely with age at which ART was started suggesting that starting ART earlier in children promotes immune recovery. This finding supports current guidance for treating HIV infection in children.^[31]^ Early and sustained ART limits the HIV reservoir and protects the CD4:CD8 ratio in adults.^[13,32,33]^ The majority of our cohort did not receive ART until several years into childhood however, likely due to delayed diagnosis and historical availability and tolerability of pediatric ART formulations. This period is similar to the period of unchecked viral replication reported in adult-acquired HIV infection prior to starting treatment (approximately eight years to a CD4 count threshold of <200 cells/μL).^[34]^ Despite this, CD4:CD8 recovery in yp-PaHIV more closely resembled the immune profile in adults where there was a maximum of six months of unchecked viremia.^[13]^ Immunological maturation in the presence of a replicating CD4-tropic virus will impact upon subsequent measures of immune recovery in perinatally acquired HIV infection, (reviewed in ^[35]^). Reduction in thymic output and diminution of the T-cell repertoire occur with age.^[36]^ In children and adolescents, preservation of thymic function may support immune recovery more predictably than in older adults and could explain the CD4:CD8 ratio recovery rate we observed.

This study has several limitations. Some data were missing from electronic clinical records limiting our ability to draw conclusions from statistical analyses. For example, data were insufficient to test if the number of years of suppressive ART was associated with CD4:CD8 ratio recovery. Furthermore causal relationships, for example between CMV coinfection or HIV suppression and CD4:CD8 ratio recovery could not be inferred in this cross-sectional study. Due to the high prevalence of CMV infection in sub-Saharan Africa, where perinatal transmission of HIV infection is more common, a CMV-uninfected group was unavailable for comparison. Our data are nonetheless generalizable to other such yp-PaHIV cohorts where CMV coinfection is widespread.

In our cohort of yp-PaHIV, we observed widespread CD4:CD8 ratio reversal reflecting ongoing immune activation and abnormality of the CD8+ T-cell compartment. Given the young age of yp-PaHIV, the significance of this finding for non-AIDS morbidity and mortality requires prospective study. Comparison with data from adult cohorts however, indicated a better-than-expected rate of CD4:CD8 ratio recovery, despite widespread CMV coinfection, that was supported by ART initiation earlier in childhood. Importantly, this is in accordance with current guidance for the treatment of paediatric HIV infection. These findings indicate immune resilience to be a feature of yp-PaHIV that should be fostered by early ART initiation.
